# Immunohistochemical expression of vitamin D receptor and forkhead box P3 in classic Hodgkin lymphoma: correlation with clinical and pathologic findings

**DOI:** 10.1186/s12885-020-07026-6

**Published:** 2020-06-08

**Authors:** Gaurav K. Gupta, Tanupriya Agrawal, Monika Pilichowska

**Affiliations:** 1 Department of Pathology and Laboratory Medicine, Tufts Medical Center, Boston, MA USA; 2 Department of Laboratory Medicine, Yale School of Medicine, 333 Cedar Street, New Haven, CT 06520 USA; 3 Department of Pathology and Laboratory Medicine, University of Rochester, Rochester, NY USA

**Keywords:** Lymphoma, Nutrition, Vitamin D, VDR, Hodgkin lymphoma, FOXP3

## Abstract

**Background:**

Expression of forkhead box P3 (FOXP3), a key regulator of T-cell function, in the tumor immune microenvironment is related to survival in classic Hodgkin lymphoma (CHL). Vitamin D receptor (VDR), a transcription factor agonists have been shown to induce FOXP3 expression in T-cells and enhance recruitment of these cells to the inflammatory sites. VDR expression is CHL has been described. However, there is no data on expression of VDR in context of quantity of FOXP3 positive cells in CHL.

**Methods:**

We examined and correlated immunohistochemical expression of VDR and FOXP3 along with clinical and pathology findings in 29 cases of CHL.

**Results:**

VDR was expressed in Hodgkin Reed-Sternberg (HRS) cells and background lymphocytes and FOXP3 was expressed in background lymphocytes. 82% of CHL cases, regardless of the subtype, expressed VDR and in majority of the cases, VDR expression was directly proportional to the quantity of FOXP3 expressing lymphocytes in the tumor microenvironment. In cases with higher clinical stage (III/IV), only 28.5% of cases diffusely expressed VDR and FOXP3 compared to 71.4% showing focal positivity. Whereas in cases with lower clinical stages (I/II), the expression pattern of VDR and FOXP3 was almost similar (41.6% diffuse versus 33.3% focal). Interestingly, focal VDR and FOXP3 expression pattern was significantly higher among males. Mixed cellularity cases showed predilection for focal VDR and FOXP3 expression (80% cases); whereas nodular sclerosis subtype had focal and diffuse VDR and FOXP3 expression patterns in similar proportion. Cases with diffuse VDR and FOXP3 expression were less likely to have bone marrow involvement. Epstein Barr virus- encoded small RNA (EBER) positive cases were predominantly focally positive (80%) for VDR and FOXP3.

**Conclusions:**

In summary, quantity of FOXP3 positive T-cells in CHL microenvironment seems to correlate with VDR expression. Clinical stage show a trend of inverse correlation with expression of VDR and quantity of FOXP3 positive T-cells. These findings suggest that VDR could be a possible prognostic and therapeutic target in CHL.

## Background

Classic Hodgkin lymphoma (CHL) is one of the most common lymphomas in the western countries, with an incidence of approximately 3 cases per 100,000 population annually [1]. It is distinctive among lymphomas for malignant cells, Hodgkin Reed-Sternberg (HRS) cells, account only few percent of total tumor tissue scattered among inflammatory background comprised of histiocytes, small lymphocytes, eosinophils and plasma cells [2]. CHL is treatd with chemotherapy or combined chemo-radiation approach and is overall highly curable as therapeutic agents have evolved over few decades [3]. However, a subset of patients (approximately 5–20%) either remain refractory to primary therapy or relapse after complete remission [4, 5]. Vitamin D belongs to the family of steroid hormones which plays crucial role in bone metabolism by regulating calcium and phosphate utilization. However, recent studies have shown that vitamin D is capable of modulating cellular proliferation and differentiation, apoptosis and angiogenesis through autocrine and paracrine mechanisms [6–8], and thus plays a role in cancer pathogenesis. The vitamin D receptor (VDR) acts as a nuclear transcription factor which forms a complex with 1,25(OH)_2_D_3,_ hormonally active form of vitamin D, and regulates expression of many genes involved in various physiological functions [9]. A recent study by Borchmann et al. provided epidemiological evidence suggesting protective role of Vitamin D in Hodgkin lymphoma [10]. However, the potential underlying mechanism for these observations remains unclear. Tumor-dominating CD4^+^ T cells play a major role in the pathophysiology of tumor. Studies of follicular lymphoma and classic Hodgkin lymphoma tumor microenvironment have shown a positive correlation between FOXP3, marker of regulatory T cells, expression and improved outcomes [11–13]. Adorini et al. previously reported that tolerogenic dendritic cells induced by treatment with VDR agonists promote both CD4^+^ CD25^+^FOXP3^+^ suppressor T cells induction as well as their recruitment to inflammatory sites [14]. Expression of VDR in Hodgkin lymphoma has been reported recently [15]. However, there is no data on expression of VDR in context of quantity of FOXP3 expressing cells in CHL tumor microenvironment. In this this study, we correlated immunohistochemical expression of VDR and FOXP3 along with clinical and pathologic findings in CHL.

## Methods

### Case selection and clinical data

Cases of CHL were identified from archives of pathology files at Tufts Medical Center and were randomly selected based on the tissue availability for histology and immunohistochemical studies. and clinical data was collected from medical records. Institutional review board of Tufts Medical Center and Tufts Health Sciences Campus approved the study. Twenty-nine cases were identified, including 21 males, 8 females, with a median age 32 (range 3–85 years). Clinical variables evaluated in this study included age, gender, hemoglobin, albumin, WBC, lymphocyte count, clinical stage (I/II vs III/IV), presence of B symptoms, bone marrow involvement, extra-nodal site involvement, response to treatment (complete remission or partial remission) and fatality.

### Histology and immunohistochemistry

Histologic sections (5 μm) were obtained from 10% formalin-fixed paraffin-embedded (FFPE) blocks and stained with hematoxylin and eosin (H&E) for microscopic evaluation. Immunohistochemistry using a mouse anti-VDR monoclonal antibody (D-6: sc13133 Santacruz Biotechnology, Dallas, Texas, USA) and anti-FOXP3 (NB600–242 Novus biologicals, CO, USA) was performed on serial 5 μm -thick FFPE tissue sections on autostainer (Ventana Medical System, Tuscon, AZ) as per the manufacturer’s instructions. Nuclear and/or cytoplasmic (VDR) and nuclear (FOXP3) immunoreactivity was assessed in all cases. Scoring was done based on percentage of positive cells 0 (no staining), 1+ (< 10%), 2+ (10–50%), 3+ (> 50%) cells and semiquantitative data (0–3+) was used for correlation analysis. Cases were categorized as either focal/absent (0/1+) or diffuse (2+/3+).

### Statistical analysis

Data were analyzed using the GraphPad Prism 8.0 software (GraphPad, San Diego, CA). Groups were compared using two-sided Fisher’s exact test and Speraman’s rank correlation test. The criterion for significance was *P*-value < 0.05.

## Results

### Clinical characteristics

Clinical features and demographics of patients are described in Table 1. The cases were comprised of 72.4% males and 27.6% females with median age 32 (range 3–85 years); median follow-up was 6 years. The age of presentation was less than 45 years in 31% cases compared to 69% cases who presented at more than 45 years of age. The primary site of involvement was mediastinum in 70.3% cases and extra-mediastinal in 29.6% cases. 51.8% of all cases showed extranodal involvement. Among all cases, 65.5% were nodular sclerosis subtype, 17.2% were mixed cellularity, 3% lymphocytes rich with, and 13.8% cases were not further subclassified. B-symptoms were present in 58.6% cases. International prognostic score (IPS) were calculated in advanced stage cases (IIB-IV); 42.8 cases had low (score 0 to 2) whereas IPS was high (3–7) in 57.8% cases. Proportion of cases with Ann Arbor stage III/IV (48.3%) was slightly more compared to Stage I/II cases (41.4%). Patient who achieved remission after first-line treatment accounted for 76, and 24% cases had persistent disease, among in which follow-up data were available. Large proportion of cases (16/20: 80%) were alive at the end of observation, among cases in which follow up data were available.

**Table 1:** Correlation of VDR and FOXP3 immunohistochemical expression and clinical data in classic Hodgkin lymphoma

Clinical Features	VDR and FOXP3 Diffuse *N*(%)	VDR and FOXP3 Focal/Absent *N*(%)	VDR Diffuse and FOXP3 Focal/Absent *N*(%)	VDR focal/absent and FOXP3 diffuse *N*(%)	Total *N*(%)	*P* Value ^a^
Age (years)						
> 45	2 (22.2)	7 (77.8)	0	0	9 (100)	0.3
≤ 45	7 (35)	10 (50)	0	3 (15)	20 (100)	
Gender						
Male	4 (19)	15 (71.4)	0	2 (9.5)	21 (100)	0.02*
Female	5 (62.5)	2 (25)	0	1 (12.5)	8 (100)	
B Symptoms						
Present	6 (35.2)	9 (52.9)	0	2 (11.7)	17 (100)	> 0.9
Absent	2 (25)	5 (62.5)	0	1 (12.5)	8 (100)	
Unknown	1 (25)	3 (75)	0	0	4 (100)	
Ann Arbor Stage						
I/IIA	4 (50)	4 (50)	0	0	8 (100)	0.4
IIB/III/IV	5 (27.7)	10 (55.5)	0	3 (16.6)	18 (100)	
Unknown	0	3 (100)	0	0	3 (100)	
IPS Score						
0–2	3 (37.5)	3 (37.5)	0	2 (25)	8 (100)	0.3
3–7	2 (18.1)	8 (72.7)	0	1 (9)	11 (100)	
Unknown	0	1 (100)	0	0	1 (100)	
Extranodal involvement						
YES	3 (21.4)	10 (71.4)	0	1 (7.1)	14 (100)	0.2
NO	6 (46.1)	5 (38.5)	0	2 (15.4)	13 (100)	
Unknown	0	2 (100)	0	0	2 (100)	
Histopathology						
NS	7 (36.8)	9 (47.4)	0	3 (15.8)	19 (100)	0.6
MC	1 (20)	4 (80)	0	0	5 (100)	
LR	1 (100)	0	0	0	1 (100)	NA
NOS	0	4 (100)	0	0	4 (100)	NA
Bone Marrow Involvement						
Yes	0	5 (100)	0	0	5 (100)	0.053
No	9 (45)	8 (40)	0	3 (15)	20 (100)	
Not evaluated	0	4 (100)	0	0	4 (100)	
EBER						
Positive	1 (20)	4 (80)	0	0	5 (100)	> 0.9
Negative	6 (30)	12 (60)	0	2 (10)	20 (100)	
Not evaluated	2 (50)	1 (25)	0	1 (25)	4 (100)	
Remission after first-line treatment						
Yes	7 (36.8)	10 (52.6)	0	2 (10.5)	19 (100)	> 0.9
No	2 (33.3)	3 (50)	0	1 (16.7)	6 (100)	
Unknown	0	4 (100)	0	0	4 (100)	
Outcome at the end of observation						
Alive	5 (31.2)	8 (50)	0	3 (18.8)	16 (100)	> 0.9
Dead	1 (25)	3 (75)	0	0	4 (100)	
Unknown	3 (33.3)	6 (66.7)	0	0	9 (100)	

Fisher’s exact test

^a^ VDR and FOXP3 diffuse vs VDR and FOXP3 focal/absent

*P value is significant (< 0.05)

*Abbreviations*:

*EBER* EBV-encoded small RNA, *FOXP3* forkhead Box P3, *LR* lymphocyte rich, *MC* mixed cellularity, *NOS* not otherwise specified, *NS* nodular sclerosis, *VDR* vitamin D receptor

### Histology and immunohistochemical characteristics

**Fig. 1:**
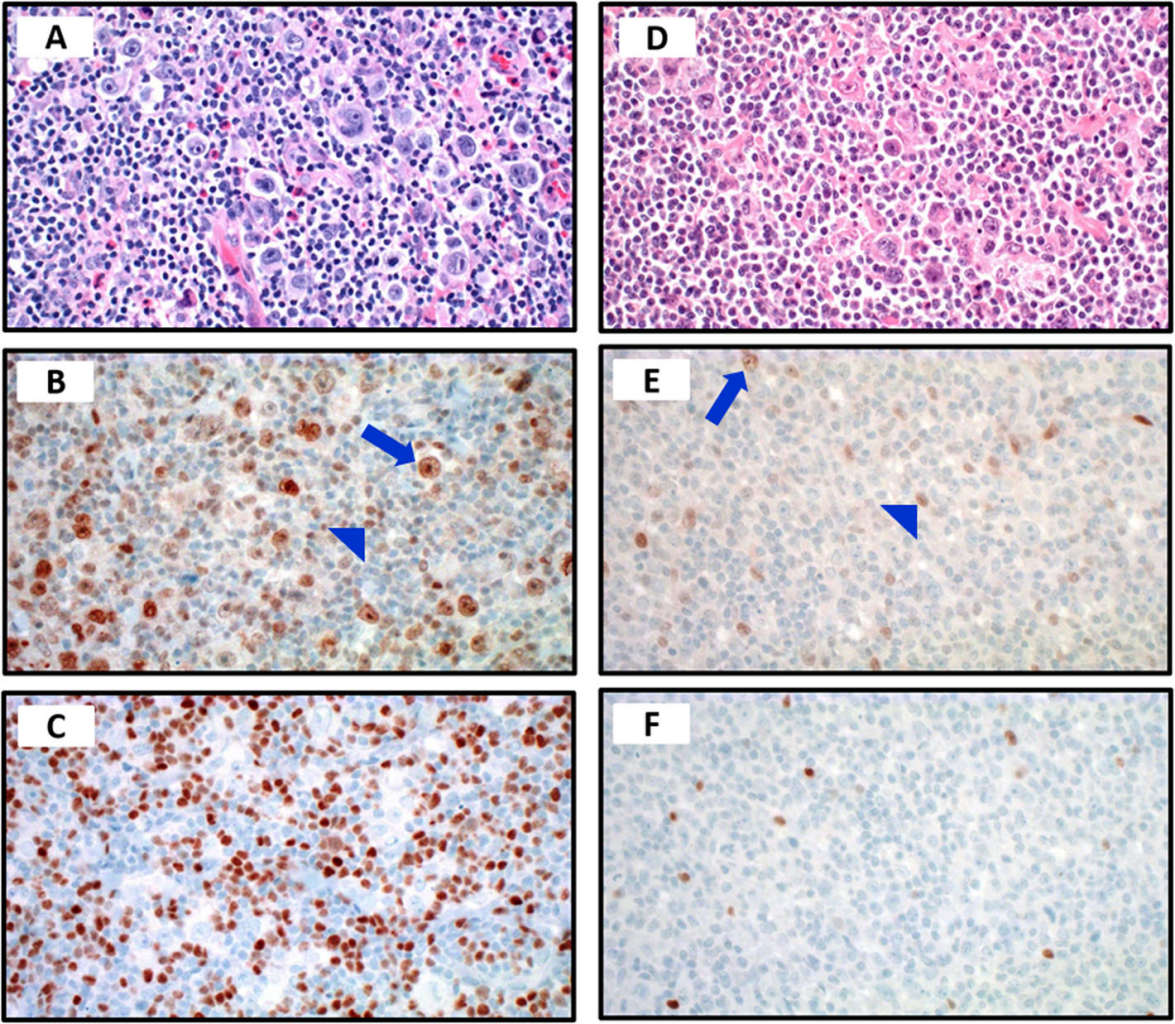
High and low VDR and FOXP3 expression patterns in CHL. Representative photomicrographs of a CHL case hematoxylin and eosin stain showing HRS and inflammatory background [**a**] with corresponding high VDR expression in HRS cells (arrow) and bystander cells (arrowhead) [**b**], and high number of FOXP3 expressing bystander cells [**c**]. Representative photomicrographs of a CHL case (hematoxylin and eosin stain) showing HRS and inflammatory background [**d**] with corresponding low VDR expression in HRS cells (arrow) and bystander cells (arrowhead) [**e**], and low number of FOXP3 expressing bystander cells [**f**]

**Fig. 2:**
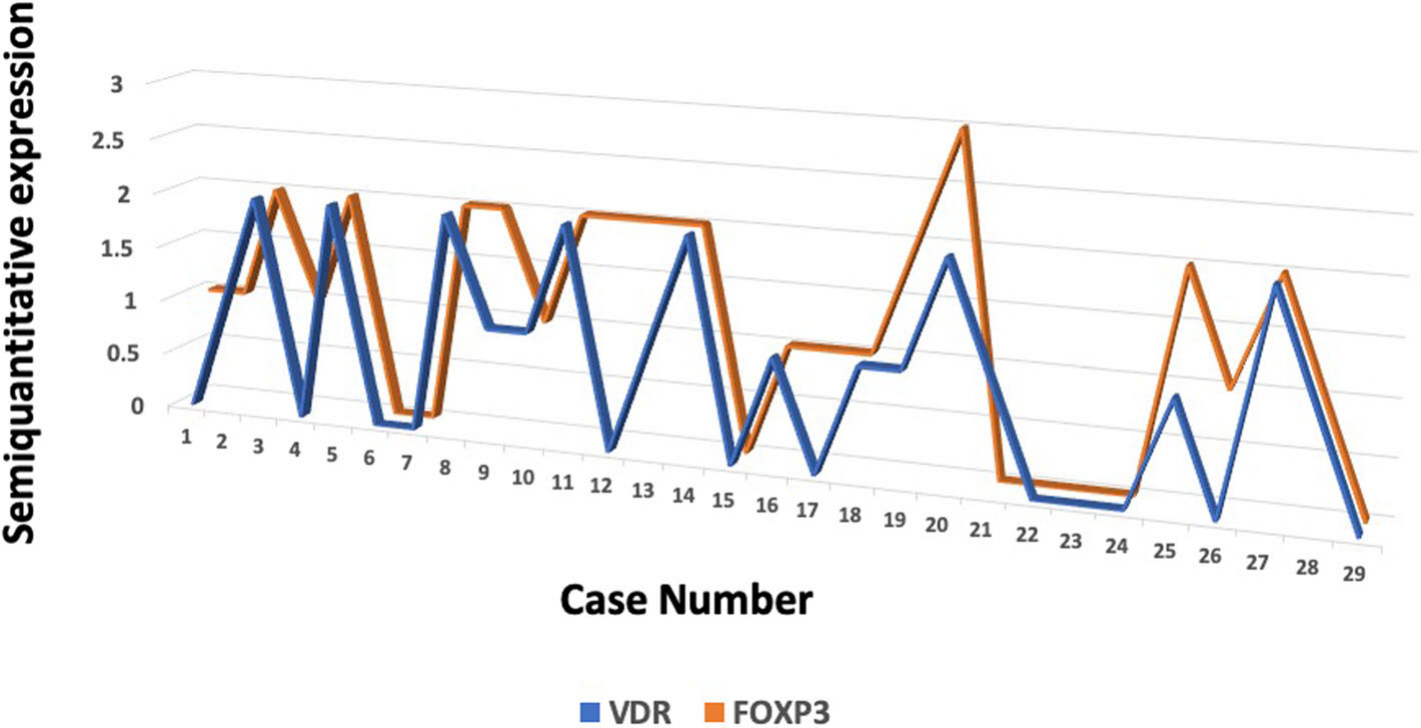
Correlation between VDR and FOXP3 expression in CHL

VDR was expressed in in the nucleus of HRS cells and bystander lymphocytes and FOXP3 was expressed in the nucleus of bystander lymphocytes (Fig. 1). The staining pattern was categorized as absent (0), focal (< 10%) or diffuse (2+/3+), based on percentage of positive cells. VDR was expressed regardless of the Hodgkin disease (HD) subtype with an overall positivity of 82% cases and FOXP3 was expressed in 78%, also regardless of HD subtype. In majority of cases VDR and FOXP3 expression pattern showed positive correlation [Fig. 2 (Spearman r 0.7431; 95% confidence interval 0.5092 to 0.8748; *P* value <.0001). The cases were divided in to four groups; VDR and FOXP3 diffuse expression (31%); VDR/FOXP3 focal/absent expression (58.6%); VDR diffuse and FOXP3 focal/absent expression (0%); VDR focal/absent and FOXP3 diffuse expression (10.3%). The data were compared among VDR and FOXP3 diffuse expression versus VDR/FOXP3 focal/absent expression groups. Interestingly, significantly higher percentage of males (71.4%) had focal VDR and FOXP3 expression versus 25% females (*P* = .02). The cases with advanced Ann Arbor stage (IIB/III/IV) showed trend towards focal VDR and FOXP3 positivity (55.5%) compared to diffuse pattern (27.7% cases). However, in lower Ann Arbor stage cases (I/II) the proportion of diffuse (50%) versus focal (50%) VDR and FOXP3 expression was similar. Among evaluated cases, all cases with bone marrow involvement were in focal/absent VDR and FOXP3 expression group versus diffuse VDR and FOXP3 expression (P .053). Mixed cellularity cases showed predilection for focal VDR and FOXP3 expression (80% cases); whereas nodular sclerosis subtype had focal and diffuse patterns in similar proportion of cases 47.4 and 36.8% respectively. Majority of cases (80%) with EBER (Epstein Barr Virus- encoded small RNA) positivity showed focal VDR and FOXP3 expression. Higher proportion of cases with fatality (75%) showed focal VDR and FOXP3 compared to the patients alive at follow up (50%).

## Discussion

In the present study, we found that VDR was found in the majority of CHL cases irrespective histologic subtype. The expression of VDR in CHL has been previously described [15] and our findings are consistent with the prior study. However, to our knowledge we are first to report clinical and pathologic correlation of VDR expression in CHL. Additionally, we also report quantity of FOXP3 expressing lymphocytes in tumor microenvironment and its correlation with VDR expression. We found that VDR expression and quantity of FOXP3 expressing lymphocytes are in a direct correlation in these cases. Majority of patient who were deceased at the end of observation period had focal to absent VDR expression and all of them had advanced stage disease. VDR was focally expressed in higher proportion of cases with advanced stage disease. These results suggest inverse correlation between disease stage and VDR expression, signifying VDR downregulation with disease progression. The inverse correlation between VDR and disease progression in skin pigmented lesion [16] and melanoma [17] has been previously described. Another recent study by Al-azhari et al. has reported inverse correlation between VDR expression and aggressive tumor characteristics in breast cancer cases [18]. Our findings show similar trends in CHL. Intriguingly, cases with diffuse VDR and FOXP3 expression were less likely to have bone marrow involvement. Bone marrow involvement in CHL has been shown to be significantly inversely proportional to overall and progression free survival and is an indicator of disseminated disease irrespective of stage [19].

Recent literature shows anti-tumor property of vitamin D [20–22] and these effects are mediated by VDR [23]. VDR acts as a transcription factor which limits tumor cells growth by affecting expression of various genes and in turn regulating various signaling pathways and cell growth [24]. Interestingly, focal VDR and FOXP3 expression was proportionately significantly high among males compared to the females. Previously, Correale et al. has shown gender difference in vitamin D immunomodulatory effects in multiple sclerosis as well as healthy subjects [25]. In this study authors demonstrated that despite similar serum vitamin D levels, upon stimulation by 1,25-(OH)2D3 (active form of vitamin D), induction of FOXP3-positive regulatory T-cells were significantly higher in among female subjects compared to males. The study suggested estrogen-promoted differences in vitamin D metabolism. However, underlying mechanism for these findings, especially in the setting of CHL, remains to be further elucidated. Recent studies have shown that increased number of FOXP3 positive cells in the tumor microenvironment favorably affect the prognosis in different lymphomas [12, 26] including CHL [27]. In a study, Jeffery et al. reported that stimulation of T-cells with active vitamin D, 1, 25 (OH)_2_D_3_ promote FOXP3 expression [28]. Kang et al. later showed that promotion of FOXP3 expression in CD4 T cells occurs due to direct bindings of VDR to vitamin D receptor binding element (VDRE) on FOXP3 gene [29]. Recently, a study by Lu et al. has shown that VDR agonist induce FOXP3 positive regulatory T cells in ulcerative colitis patients [30]. In our study we have observed direct correlation between VDR expression and quantity of FOXP3 positive lymphocytes in the tumor microenvironment suggesting a possibility of immunomodulatory role of VDR in the CHL tumor microenvironment consistent with in vitro findings in above mentioned studies. Additionally, our results showed that cases with EBER positivity had lower VDR expression. In a previous study Yenamandra et al. has shown that infection of human B-cells with Epstein Barr Virus (EBV) cause downregulation of VDR gene [31]. Results from another in vitro study showed that VDR mRNA and protein expression is decreased in a multiple myeloma cells line by EBV [32]. Therefore, our findings support previous observations from in vitro studies raising a possibility of EBV and VDR interaction, with EBV downregulating VDR in CHL cases. Interestingly, we have observed that MC subtype of CHL cases had overall lower VDR expression. Mixed cellularity subtype of CHL are known to be more frequently EBV-positive (approximately 75%) than nodular sclerosis subtype (10–25%) [33], as in our study.

## Conclusions

In summary, our data demonstrate that VDR and FOXP3 are expressed in CHL and that there is a direct correlation between VDR expression and quantity of FOXP3 expressing lymphocytes in CHL tumor microenvironment. In addition, there is a trend for inverse correlation between clinical stage and VDR and FOXP3 expression. Significantly high proportion of males show focal VDR and FOXP3 expression. Cases with diffuse VDR and FOXP3 expression were less likely to have bone marrow involvement. Therefore, combination of VDR and FOXP3 immunohistochemical staining could aid prognostication in CHL. Since VDR ligands positively influence VDR expression [34] potential therapeutic importance for Vitamin D in CHL needs to be considered. However, additional studies are needed to identify underlying mechanism, likely multifaceted, for these findings.

## Data Availability

The datasets used and/or analyzed during the current study are available from the corresponding author on reasonable request.

## Abbreviations

CHL: Classic Hodgkin lymphoma
FOXP3: Forkhead Box P3
EBV: Epstein Barr Virus
HRS Cell: Hodgkin Reed-Sternberg cell
IPS: International prognostic score
VDR: Vitamin D receptor
